# WHCNS Model-Based Quantitative Analysis of Water and Nitrogen Use Efficiencies and Environmental Benefits in Chernozem Farmland

**DOI:** 10.3390/plants15213357

**Published:** 2026-10-29

**Authors:** Liang Jin, Dan Wei, Qilin Lv, Dawei Yin, Qiang Zuo, Jianli Ding, Yan Li, Yu Hu, Lina Liang, Lei Sun, Wei Wang, Huibo Shen, Yuxian Wang, Xu Shang, Nicholas John Clarke, Lei Wang

**Affiliations:** 1 Institute of Plant Nutrition, Resources and Environment, Beijing Academy of Agricultural and Forestry Sciences, Beijing 100097, China; jinliang19762003@aliyun.com (L.J.); lv_qilin_lql@163.com (Q.L.); zq18189@163.com (Q.Z.); lndjl@126.com (J.D.); huyu0805@126.com (Y.H.); lianglinacau@163.com (L.L.); 2 College of Agricultural Science and Technology, Heilongjiang Bayi Agricultural University, Daqing 163319, China; yindazhiyindawei@126.com (D.Y.); shangxu413@163.com (X.S.); 3 Heilongjiang Black Soil Protection and Utilization Research Institute, Heilongjiang Academy of Agriculture and Sciences, Harbin 100094, China; li.yan622@163.com (Y.L.); tufeisuosunlei@163.com (L.S.); wangwei123873@163.com (W.W.); 4 Qiqihar Branch Institute, Heilongjiang Academy of Agricultural Sciences, Qiqihar 161005, China; shenhuibo@163.com (H.S.); 13836209740@163.com (Y.W.); 5 Norwegian Institute of Bioeconomy Research, P.O. Box 115, 1431 Ås, Norway; nicholas.clarke@nibio.noi

**Keywords:** mollisols, agroecosystem modelling, spring maize, straw return, organic manure substitution, ammonia volatilisation, denitrification, semi-arid region

## Abstract

Semi-arid Chernozem farmland in Northeast China faces the dual challenges of water scarcity and excessive nitrogen (N) fertilisation, yet quantitative understanding of coupled water–N use efficiency and environmental trade-offs under climatic variability remains limited. Using the WHCNS (Water, Heat, Carbon and Nitrogen Simulator) model calibrated and validated against field data (2022–2023) from Qiqihar, we quantified crop yield, water use efficiency (WUE), System-Level Nitrogen Conversion Efficiency (SLNCE) and N losses under five management regimes encompassing conventional fertilisation, straw mulching and organic manure substitution. Long-term climate adaptability was evaluated using precipitation scenarios from 2001 to 2023. Straw mulching reduced evapotranspiration by 12.2% and increased WUE to 2.09 kg·m^−3^. Combined chemical and organic fertiliser application (T223) achieved the highest yield (9745.9 kg·ha^−1^) and WUE (2.14 kg·m^−3^), whereas a 30% reduction in chemical fertiliser with straw return (T323) reduced yield and SLNCE, indicating insufficient N supply. Organic substitution achieved the highest SLNCE (35.9 kg·kg^−1^), exceeding the straw-only treatment by 29.1%. Nitrate leaching below 1 m was negligible, whereas gaseous N losses were the dominant loss pathway and increased with rainfall. SLNCE responded nonlinearly to precipitation, peaking at 420–451 mm; extreme drought years constrained yield through nitrogen limitation, whereas wet years increased gaseous N losses while maintaining relatively high yields. These results show that organic substitution can simultaneously enhance WUE and SLNCE, providing a climate-adaptive strategy for sustainable water–N management in semi-arid Chernozem soils.

## 1. Introduction

Global climate change has increased the frequency of extreme weather events, creating substantial challenges for agricultural production [1]. Recent CMIP6-based projections indicate continued warming across China under all SSP scenarios, accompanied by spatially heterogeneous changes in precipitation [2]. The black soil region of Northeast China contributes approximately one-fourth of the national commodity grain supply; however, drought and heatwaves have intensified under the combined effects of global warming and regional climate variability. Drought is a recurrent threat to spring maize across the Songnen Plain and may become more frequent in the coming years [3]. Under water-limited conditions, intensive nitrogen inputs intended to sustain high yields can increase nitrate leaching and diminish the environmental benefits of maize production [4].

Process-based models provide a robust framework for quantitatively examining these challenges. Since the 1960s, agricultural systems modelling has progressed from empirical approaches to process-based and modular crop-system models, including APSIM and DSSAT [5,6]. Among the available models, the WHCNS (i.e., Water, Heat, Carbon and Nitrogen Simulator) model integrates modules from RZWQM, HYDRUS-1D and DAISY and can simulate soil water, heat, carbon and nitrogen processes together with their coupling with crop growth [7]. The model has been preliminarily validated in the black soil region of Northeast China. In Lishu County, WHCNS simulations produced a multi-year average spring maize yield of 8861–12,508 kg·ha^−1^ and total nitrogen losses of 25–78 kg·ha^−1^. Simulated maize yield was significantly influenced by precipitation, solar radiation and soil clay content [8]. These findings support the applicability of the model in this region [9].

Nevertheless, most regional simulation studies have focused on winter wheat–summer maize rotation systems in the North China Plain and rice-growing regions of southern China. Systematic simulation of farmland water–nitrogen coupling in the semi-arid Chernozem soils of Northeast China remains relatively limited [10,11]. Previous studies have mainly examined optimal combinations of water and fertiliser management; consequently, the synergy among water and nitrogen use efficiencies and environmental benefits in semi-arid regions remains insufficiently quantified [12].

In this study, typical Chernozem spring maize farmland in the Qiqihar area of Heilongjiang Province was selected to calibrate and validate the applicability of the WHCNS model using field positioning experimental data. We then established multilevel water and nitrogen management scenarios to quantify their effects on maize yield, water and nitrogen use efficiencies and environmental nitrogen loss. The study tested the following hypotheses: (1) reducing chemical fertiliser in combination with organic fertiliser improves the spatiotemporal matching of nitrogen supply and demand through slow nutrient release, thereby enhancing nitrogen use efficiency; and (2) straw mulching and incorporation reduce evapotranspiration by decreasing soil surface evaporation, thereby increasing water use efficiency. By quantitatively evaluating the effects of different water and nitrogen management regimes on crop yield, water and nitrogen use efficiencies and environmental nitrogen loss, this study aims to support improved scientific decision-making for water and nitrogen management in the semi-arid Chernozem region of Northeast China. In cold, semi-arid climates, organic fertiliser mineralisation is governed by microbial community composition, temperature sensitivity and moisture availability, which together determine the rate and timing of nitrogen release; therefore, the slow-release effect assumed in the first hypothesis depends on the balance between microbial immobilisation and mineralisation under low-temperature and water-limited conditions.

## 2. Results

### 2.1. Calibration of WHCNS Model Parameters

The soil–crop system model contains numerous parameters, making calibration inherently complex. Sensitivity analysis can quantify the influence of individual parameters on model outputs and thereby support parameter optimisation [13]. Parameters with low sensitivity can be fixed to simplify model inputs, whereas highly sensitive parameters can be prioritised during calibration, thereby improving model calibration efficiency and accuracy [14].

**Figure 1:**
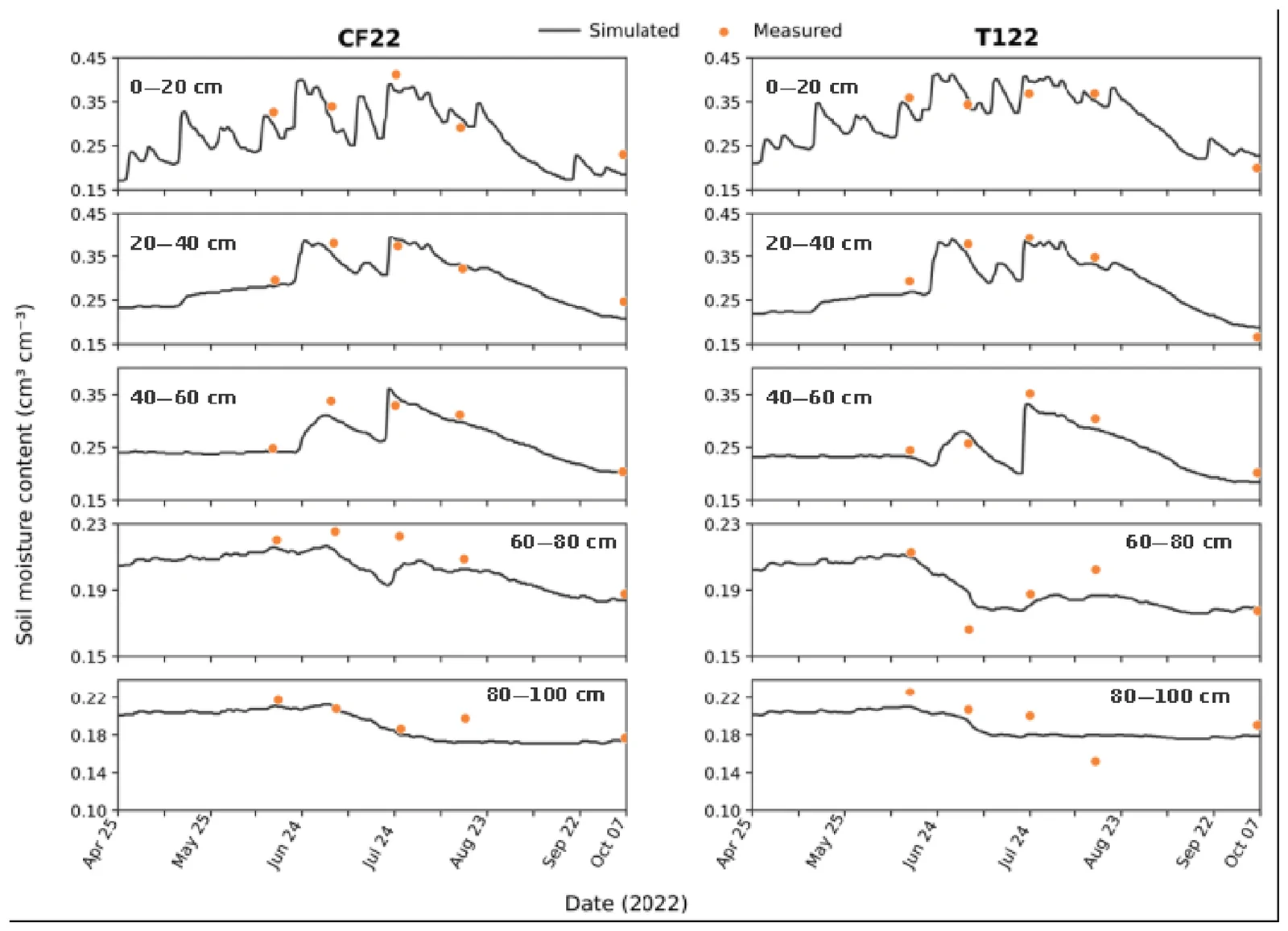
Comparison of simulated and measured soil moisture content for all treatments in 2022.

**Figure 2:**
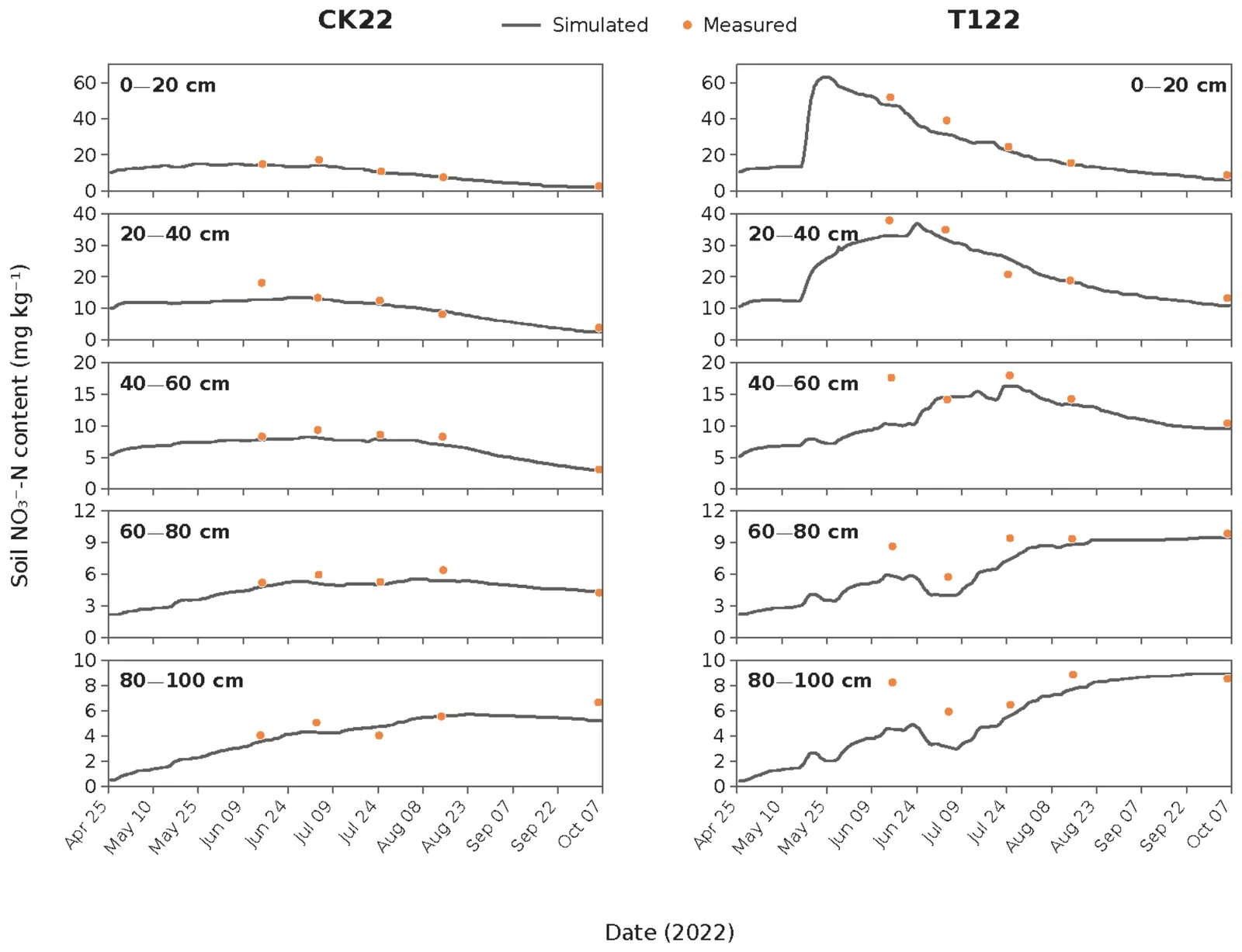
Comparison of simulated and measured soil NO_3_^-^ content for all treatments in 2022.

**Figure 3:**
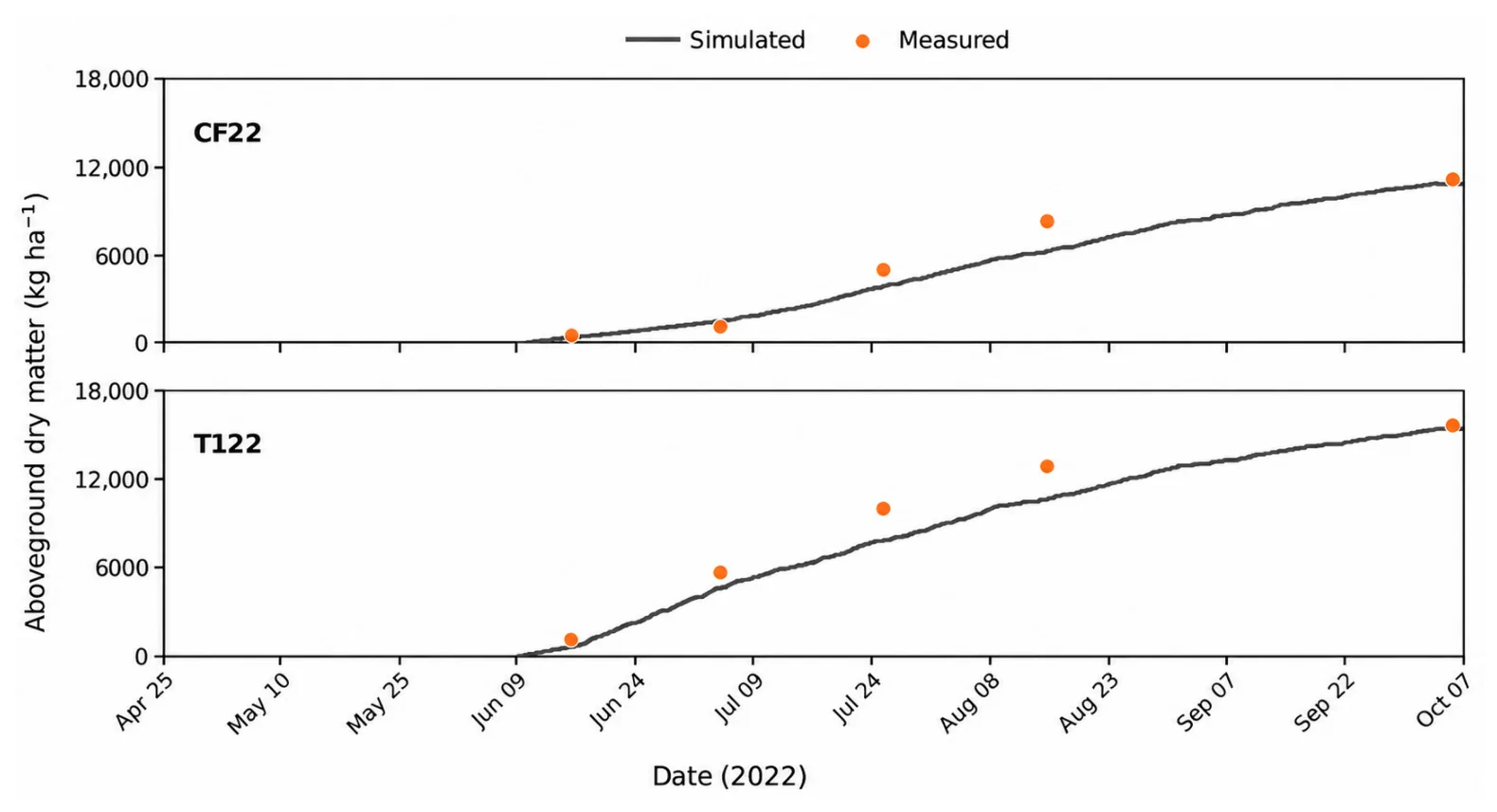
Comparison of simulated and measured aboveground dry matter mass for the CF22 and T122 treatments in 2022.

**Figure 4:**
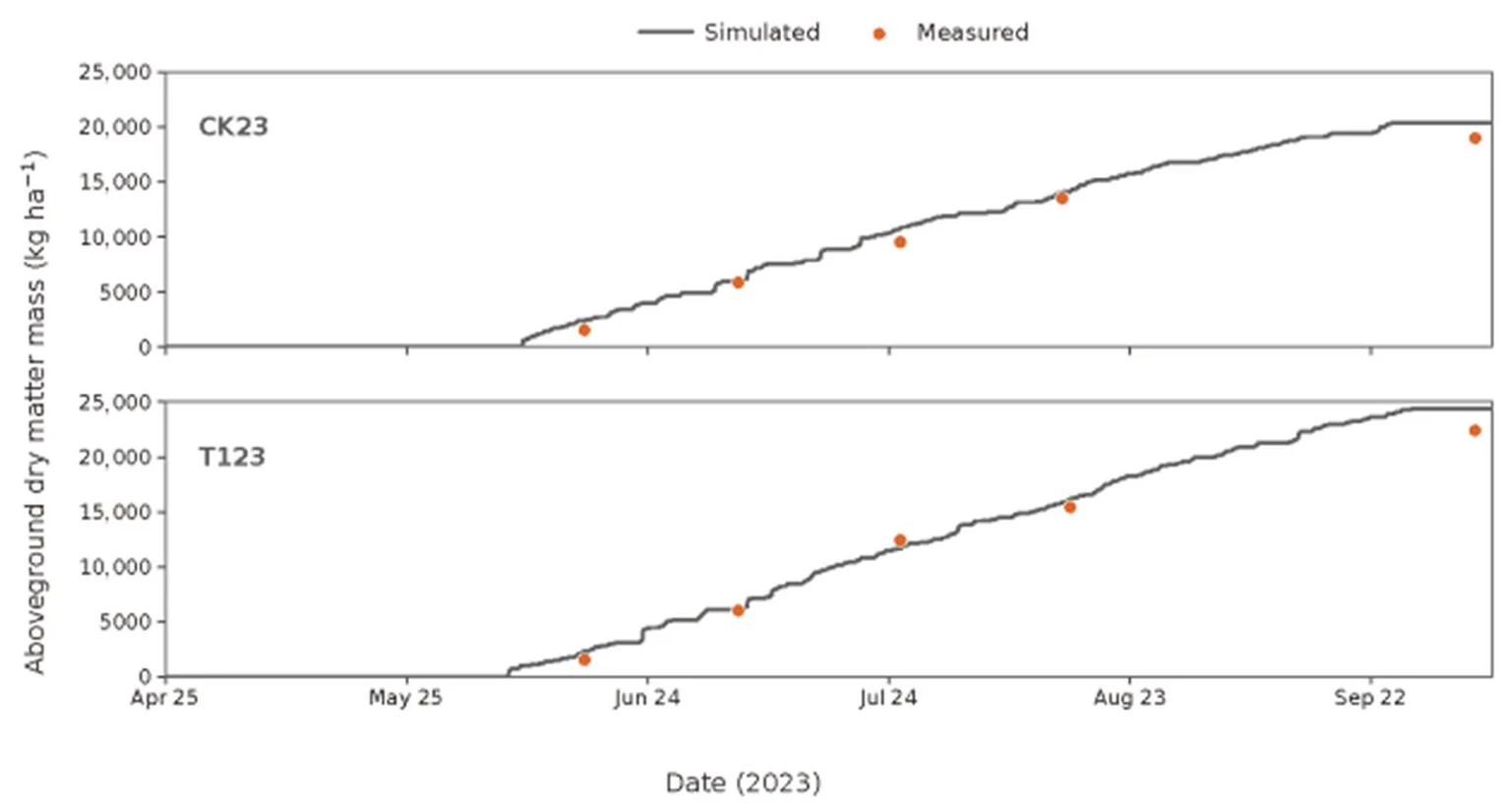
Comparison of simulated and measured aboveground dry matter mass for the CK23 and T123 treatments in 2023.

For sensitivity analysis, data from the CF22 treatment at the Qiqihar experimental site were used, and individual parameters were varied using a single-factor perturbation method within ±10% while all other parameters were held constant. The resulting effects on soil water content (i.e., 0–100 cm), nitrate–nitrogen content and aboveground dry matter mass were systematically evaluated (Figure 1, Figure 2, Figure 3 and Figure 4), and sensitivity coefficients were calculated for each parameter. All calibrated parameter values remained within their respective reasonable ranges (Table 1 and Table 2).

**Figure 5:**
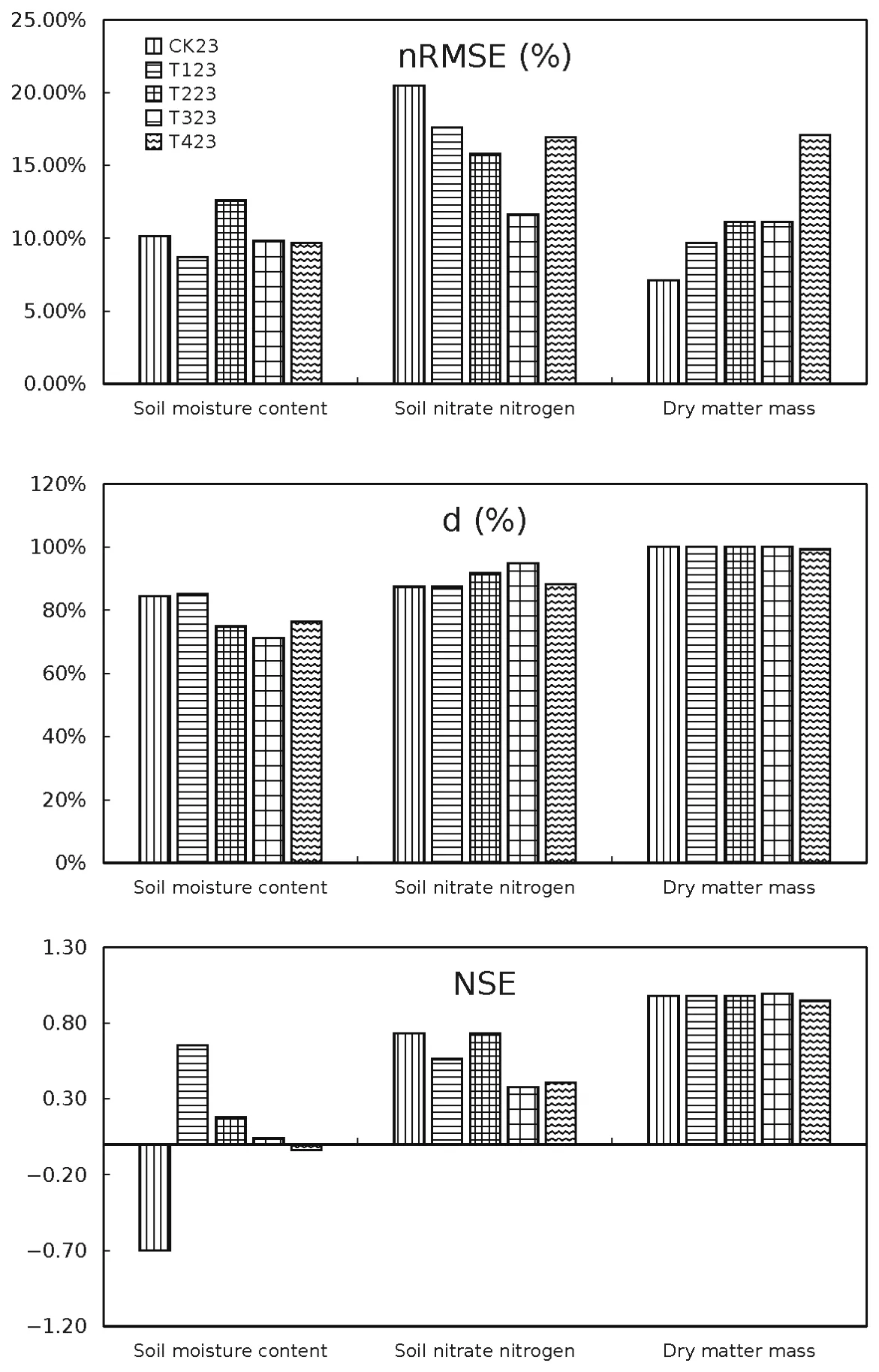
Model simulation performance for soil water content, nitrate–nitrogen content and aboveground dry matter mass in 2023.

As shown in Figure 3 and Figure 4, the simulated aboveground dry matter mass for the CF22 and T122 treatments in 2022 and for the CK23 and T123 treatments in 2023, after calibration, agreed well with the measured values. During calibration (Figure 5), nRMSE for soil moisture was 5.77–10.26%, with NSE values of 0.16–0.94. For nitrate–nitrogen content, nRMSE was 11.52–20.32% (i.e., 0–60 cm depth), with NSE values of 0.41–0.92. For aboveground dry matter mass, nRMSE was 13.63–20.69% and ranged from 0.98 to 0.99. These results demonstrate good model applicability in the study area.

The preceding results show good agreement between measured and simulated values from the 2023 fertiliser application experiments. Thus, the WHCNS model effectively simulates maize growth and soil water and nitrogen dynamics under both straw return and reduced chemical fertiliser application combined with straw return.

### 2.2. WHCNS-Based Model Simulation of Water and Nitrogen Balance in Maize Farmland During Straw Return

As shown in Table 3 and Table 4, total precipitation during the growth period of spring maize in 2023 reached 579.5 mm. However, due to spring drought, the 90 mm of supplemental irrigation applied after sowing was necessary to ensure seedling establishment. The complete water balance (Table 3) shows that crop water consumption was met by the combined supply of precipitation and supplemental irrigation. Relative to the CK23 treatment with full chemical fertiliser application (i.e., evapotranspiration of 513.9 mm), evapotranspiration decreased to 451.3 mm in T123 and to 454.8 mm, 459.8 mm and 460.8 mm in T223, T323 and T423, corresponding to reductions of 12.18%, 11.50%, 10.53% and 10.33%, respectively. Crop yield under T223 was 24.09% higher than that under T323 and 7.79% higher than that under T423. No significant differences in crop yield were observed among CK23, T123 and T223; however, yields under all three treatments were markedly higher than those under T323. WUE followed the order T223 > T123 > T423 > CK23 > T323. The WUE of T223 was 15.05% higher than that of CK23, indicating that chemical fertiliser application combined with organic fertiliser decreases evapotranspiration while maintaining a relatively high crop yield, thereby significantly enhancing WUE.

Table 5 presents the simulated soil nitrogen dynamics and SLNCE under the different fertilisation treatments in 2023. Crop nitrogen uptake accounted for 61.5–80.1% of total nitrogen output (i.e., crop uptake + ammonia volatilisation + denitrification + leaching loss). T123 had the highest crop nitrogen uptake (i.e., 260.5 kg N·ha^−1^), exceeding the other treatments by 19.5–49.8%, whereas T323 had the lowest uptake (i.e., 173.9 kg N·ha^−1^). Ammonia volatilisation was highest under T323 (i.e., 75.4 kg N·ha^−1^) and lowest under T423 (i.e., 35.3 kg N·ha^−1^). The highest denitrification loss occurred under T123 (i.e., 41.1 kg N·ha^−1^), significantly exceeding the losses under the other treatments (i.e., 14.8–16.6 kg N·ha^−1^). The model simulated zero nitrogen leaching loss below the 1 m soil layer, indicating that nitrate–nitrogen leaching into the deeper soil layer was not simulated.

T423 achieved the highest SLNCE, which was 5.6% higher than that of CK23. At a nitrogen application rate of 195 kg·ha^−1^, SLNCE under T223 was 1.5% and 3.7% higher than under CK23 and T123, respectively. At a nitrogen application rate of 136.5 kg·ha^−1^, SLNCE under T423 (i.e., 70% chemical fertiliser + organic fertiliser) was 29.1% higher than under T323 (i.e., 70% chemical fertiliser + straw return).

Table 6 presents the WHCNS-simulated soil nitrogen dynamics (i.e., ammonia volatilisation, crop uptake, leaching loss and denitrification) and SLNCE within the 1 m soil layer under different precipitation-year scenarios. For T323 in each precipitation-year type, nitrogen output followed the order crop uptake > ammonia volatilisation > denitrification > leaching loss, with crop uptake representing the dominant pathway and accounting for 54.20–77.52% of total nitrogen output. Nitrogen leaching loss remained zero in all years; thus, the model simulated no nitrate–nitrogen leaching below the 1 m soil layer in the semi-arid Chernozem region, regardless of precipitation-year type. As annual precipitation increased from 206.7 mm to 579.5 mm, denitrification loss increased markedly (i.e., from 0.2–1.1 kg·ha^−1^ to 33.6–43.8 kg·ha^−1^), while ammonia volatilisation concurrently increased (i.e., from 34.4–46.0 kg·ha^−1^ to 75.4–86.4 kg·ha^−1^). Accordingly, total gaseous nitrogen loss through denitrification and ammonia volatilisation increased significantly with increasing precipitation.

Under identical precipitation conditions, gaseous nitrogen loss under T323 (i.e., 70% compound fertiliser + straw return) was lower than under T122, indicating that reduced chemical fertiliser application can effectively decrease gaseous nitrogen loss. Overall, T323 maintained a high SLNCE while also significantly reducing gaseous nitrogen loss.

## 3. Discussion

### 3.1. Regulatory Effects of Straw Mulching and Reduced Chemical Fertiliser Application on Evapotranspiration

Model parameter sensitivity is strongly influenced by the climatic, soil and field management conditions of the study area [11,13,14]. Consequently, parameters calibrated for Qiqihar may differ from those reported for other regions, underscoring the need for local calibration before applying WHCNS at new sites. Both straw returning and reduced chemical fertiliser application suppressed evapotranspiration (ET) by 10–12%, although through fundamentally different regulatory pathways (Table 4).

Straw mulching and incorporation can reduce soil surface evaporation through combined physical and biological effects. The straw layer intercepts direct solar radiation, moderates soil-surface temperature fluctuations and reduces near-surface wind velocity, thereby disrupting hydraulic continuity at the soil–atmosphere interface and suppressing evaporation through water phase transition [15]. In semi-arid regions, soil evaporation can account for a substantial proportion of crop evapotranspiration, and mulching can markedly reduce this non-productive water loss [16]. Consequently, straw mulching and incorporation have considerable water-saving potential. In this study, T123 had the lowest evapotranspiration (i.e., 451.3 mm), representing a 12.18% reduction relative to CK23 and confirming this effect. In addition, humus formed during straw decomposition can improve soil aggregate structure and increase soil water retention, with particularly pronounced long-term effects during the middle and late crop growth stages [17,18].

The reduction in evapotranspiration associated with reduced chemical fertiliser application is primarily attributable to optimisation of canopy structure. Excessive nitrogen fertilisation can promote excessive vegetative growth, and an overly high leaf area index can increase luxury water consumption through canopy transpiration [19]. In this study, CK23 had the highest evapotranspiration (i.e., 513.9 mm), but not the highest crop yield, indicating a relatively high proportion of ineffective transpiration within total water consumption. By contrast, the T423 treatment can balance vegetative and reproductive growth through reduced chemical fertiliser inputs, whereas the T223 treatment optimises the source–sink relationship through the synergistic supply of chemical and organic fertilisers. This interpretation is consistent with findings for winter wheat–spring maize rotations in semi-arid regions reported by Liu et al. [12], who showed that optimised nitrogen application can improve crop water relations and enhance transpiration efficiency. The evapotranspiration of T323 (i.e., 70% chemical fertiliser + straw return) was 459.8 mm, slightly higher than that of T123 and T223, although the differences were not significant. This result suggests that, under reduced chemical fertiliser conditions, the physical mulching effect of straw return and the physiological regulation associated with organic fertiliser may be functionally complementary; however, their synergistic optimisation requires further investigation.

### 3.2. Synergistic Regulation of Water–Nitrogen Coupling on Crop Yield and WUE

The combination of high yield and high WUE under the combined chemical–organic treatment (T223) indicates that water–N optimisation depends on decoupling water consumption from yield formation rather than simply minimising total water use. This enables the dual benefit of reduced water consumption and stable nutrient uptake.

From a water–nitrogen coupling perspective, the advantages of T223 can be attributed to three factors: (1) the combined application of chemical and organic fertiliser avoids excessive inorganic nitrogen concentration in the root zone soil solution, thereby reducing osmotic stress and improving root water uptake; (2) the slow-release effect of organic fertiliser improves the synchrony between soil nitrogen supply and crop nitrogen demand; (3) the higher SLNCE under reduced chemical fertiliser application combined with organic fertiliser can be attributed to the slow-release effect of organic fertiliser, which improves synchrony between soil nitrogen supply and crop nitrogen demand [20,21]. This mechanism is consistent with the nutrient synchrony theory proposed by Crews and Peoples [20], which identifies asynchrony between nitrogen supply and crop demand as a primary cause of nitrogen loss in agricultural ecosystems; and (3) straw mulching improves root-zone soil moisture, facilitating nutrient mass flow toward the root surface. Kramer et al. [21] found that combined organic and inorganic fertiliser application improves the spatiotemporal matching of nutrient supply through a complementary mechanism: chemical fertilisers rapidly satisfy early-stage nutrient demand, whereas slow-release organic fertilisers progressively meet nutrient requirements during the middle and late growth stages. In this study, WUE under T223 was 15.05% higher than under CK23, providing a specific manifestation of this mechanism in terms of water use efficiency.

In contrast, T323 produced the lowest crop yield (i.e., 7854.0 kg·ha^−1^) and WUE (i.e., 1.71 kg·m^−3^). Although chemical fertiliser application was reduced under this treatment, it relied on straw return without supplemental organic fertiliser, resulting in insufficient soil nitrogen supply and delayed nitrogen release. Consequently, nitrogen availability did not meet maize demand during the critical growth period. This result supports the view reported by Pang and Letey [22] that reliance solely on organic nitrogen sources is insufficient to meet peak nitrogen demand in high-nitrogen crops such as maize, whereas combined organic and inorganic fertiliser application is important for achieving nutrient synchrony.

### 3.3. Evaluation of Nitrogen Dynamics and Environmental Benefits

In this study, simulated nitrogen leaching below the 1 m soil layer remained zero for all treatments, suggesting an extremely low risk of deep nitrate leaching under the modelled conditions in the semi-arid Chernozem region. However, WHCNS primarily simulates matrix flow and does not explicitly represent preferential flow through shrinkage cracks, which are common in clayey Chernozem soils during drying–rewetting cycles. Therefore, the simulated absence of nitrate leaching below 1 m may partly reflect this model simplification, and episodic deep percolation through macropores during extreme rainfall events cannot be completely excluded. This pattern is consistent with regional water limitation because precipitation (i.e., 579.5 mm) was lower than potential evapotranspiration. The negligible simulated nitrate leaching below 1 m can be attributed to the relatively low precipitation and strong water-holding capacity of Chernozem soil in this region [9,23]. Consequently, gaseous nitrogen loss (i.e., ammonia volatilisation + denitrification) was the dominant nitrogen-loss pathway, accounting for 19.9–38.5% of total nitrogen output. Ammonia volatilisation alone accounted for 12.2–26.7% of total nitrogen output, making it the largest individual nitrogen-loss pathway. T323 had the highest ammonia volatilisation (i.e., 75.4 kg N·ha^−1^), likely because of its relatively high soil pH and the asynchrony between nitrogen release and crop demand during early straw decomposition. By contrast, T423 had the lowest ammonia volatilisation (i.e., 35.3 kg N·ha^−1^), suggesting that organic fertiliser may promote ammonium retention by soil microorganisms, thereby lowering NH_4_^+^–N concentrations in the soil solution and reducing substrate availability for ammonia volatilisation [24]. The relatively high cation exchange capacity and organic matter content of Chernozem soil further favour ammonium retention and microbial immobilisation, thereby limiting NH_4_^+^–N concentrations in soil solution and ammonia volatilisation. However, the slightly alkaline soil pH shifts the NH_4_^+^ ⇌ NH_3_ equilibrium toward ammonia, partially offsetting this retention effect. For denitrification, high organic matter content supplies abundant carbon to denitrifiers, while the clayey texture promotes anaerobic microsites; together with episodic wetting–drying cycles under semi-arid conditions, these properties explain the pulse-like denitrification losses observed in wet years. The highest denitrification loss (41.1 kg N·ha^−1^) occurred under T123 and was significantly greater than under the other treatments (i.e., 14.8–16.6 kg N·ha^−1^). Straw mulching may create stronger anaerobic microenvironments during the middle and late growth stages and thereby promote denitrification.

### 3.4. Enhancement Mechanism and Optimisation Pathways for Nitrogen Use Efficiency

Organic substitution under reduced chemical fertiliser application achieved the highest SLNCE by improving nitrogen supply–demand synchrony, consistent with the nutrient synchrony theory of Crews and Peoples [20]. This advantage is associated with the slow-release nutrient effect of organic fertiliser, which improves the alignment between soil nitrogen supply and crop nitrogen demand. Moreover, Crews and Peoples [20] identified asynchrony between nitrogen supply and crop demand as a primary cause of nitrogen loss in agricultural ecosystems. In this study, T423 provided complementary nitrogen supply through the combination of 70% chemical fertiliser and organic fertiliser: chemical fertiliser can support rapid crop establishment during the seedling stage, whereas mineralisation of organic fertiliser can supplement nitrogen during the middle and late growth stages. Wang et al. [25] similarly reported in maize that 30% organic substitution can maximise plant nitrogen uptake and SLNCE by synchronising nitrogen supply with crop growth. In the present study, crop nitrogen uptake under T423 was 201.8 kg N·ha^−1^, lower than that under T123 (i.e., 260.5 kg N·ha^−1^); however, SLNCE was higher, indicating a more efficient regime characterised by relatively high nitrogen output at relatively low nitrogen input. Two relationships are particularly relevant for optimisation: (1) T223 achieved the highest WUE, whereas its SLNCE (i.e., 34.5 kg·kg^−1^) was slightly lower than that of T423 (i.e., 35.9 kg·kg^−1^), indicating a threshold effect in the synergistic optimisation of water and nitrogen use efficiencies; and (2) T123 achieved relatively high crop yield and nitrogen uptake but a comparatively low SLNCE (i.e., 30.8 kg·kg^−1^), indicating that high-input management produced high yield at a relatively substantial nitrogen environmental cost. Future studies should therefore establish multi-objective functions for jointly optimising water and nitrogen use efficiencies by integrating crop yield, WUE, SLNCE and environmental loss to identify optimal water and nitrogen management strategies for different precipitation types.

### 3.5. Interannual Variation Characteristics and Climate Adaptability Based on a Scenario Analysis

Quadratic regression of simulated SLNCE against annual precipitation in 2023 confirmed a significant unimodal response: SLNCE = −0.000185 P^2^ + 0.1913 P − 23.12 (R^2^ = 0.868, *p* = 0.017), with the fitted curve peaking at approximately 518 mm and the 95% confidence band. The unimodal response of SLNCE to precipitation indicates contrasting constraints among precipitation-year types: drought restricts N mineralisation and N supply, whereas wet conditions intensify denitrification. Thus, a single static fertilisation regime is unlikely to be optimal under climatic variability, because limited nitrogen mineralisation can constrain N supply in drought years, while enhanced denitrification increases gaseous N loss in wet years.

Denitrification loss was higher in 2022 at 43.8 kg N·ha^−1^ (i.e., precipitation of 514.8 mm) than in 2023 at 33.6 kg N·ha^−1^ (i.e., precipitation of 579.5 mm), indicating that denitrification is governed not only by total precipitation but also by its seasonal distribution, soil temperature and antecedent soil moisture. The nonlinear response of SLNCE to precipitation, with SLNCE increasing up to approximately 420–451 mm and declining in wetter years (as supported by the significant quadratic regression, R^2^ = 0.868, *p* = 0.017), indicates that both drought and wet years constrain nitrogen use efficiency. In addition, Guo et al. [26] reported that rapid soil drying–rewetting under flash drought can trigger pulsed denitrification. In this study, a similar dry–wet alternation pattern in 2022 may have caused an earlier denitrification peak. Therefore, under extreme agricultural climatic conditions, water and nitrogen management in semi-arid regions should shift from empirical judgement toward intelligent decision-making based on precise regulation systems supported by real-time monitoring of soil moisture and nitrogen.

## 4. Materials and Methods

### 4.1. Overview of the Experimental Sites

Field experiments were conducted during 2022–2023 in the typical Chernozem region of Qiqihar City, Heilongjiang Province (i.e., 47°22′ N and 123°55′ E). The study area has a temperate continental semi-arid monsoon climate, with a multi-year mean temperature of 3.2 °C, an effective accumulated temperature (≥10 °C) of 2560 °C, a multi-year mean precipitation of 422 mm, an annual evapotranspiration of 1650 mm and a frost-free period of ~132 days. The experimental soil was a typical Calcic Chernozem developed from loess-like sediment. The topsoil had a pH of 8.16, an organic matter (OM) content of 39.5 g·kg^−1^, available potassium (AK) of 158.6 g·kg^−1^, available phosphorus (AP) of 33.7 g·kg^−1^ and alkali-hydrolyzable nitrogen (AN) of 130.5 g·kg^−1^.

### 4.2. Experimental Design

The maize cultivar Nendan 19 (Hei Shen Yu 2017012; bred by the Qiqihar Branch of Heilongjiang Academy of Agricultural Sciences, Qiqihar 161006, China)was planted on ridges at a row spacing of 65 cm and a plant spacing of 25 cm, corresponding to a density of 60,000 plants·ha^−1^. Irrigation was supplied through shallow-buried drip irrigation without a membrane, with supplemental irrigation scheduled according to soil moisture status and crop water requirements during the growing period. Supplemental irrigation was initiated when soil water content in the 0–40 cm layer declined to 60% of field capacity and was stopped when soil water content reached 85% of field capacity. The total irrigation depth was 120 mm in 2022 and 90 mm in 2023, applied in 3 and 2 events, respectively. A randomised block design with 39 m^2^ plots and three replicates was used in both years. In 2022, straw return was used as the regulatory factor, and four treatments were established: (1) CF22, no fertilisation; (2) T122, compound fertiliser (N–P_2_O_5_–K_2_O = 26–10–12, Heilongjiang Wufu Min’an Agricultural Technology Co., Ltd., Qiqihar, China) at 750 kg·ha^−1^; (3) T222, T122 + full straw return; and (4) T322, humic acid fertiliser (N–P_2_O_5_–K_2_O = 29–6–9, Heilongjiang Wufu Min’an Agricultural Technology Co., Ltd., Qiqihar, China) at 450 kg·ha^−1^ + full straw return. In 2023, chemical fertiliser reduction with organic fertiliser application was used as the regulatory factor, and five treatments were established: (1) CK23, compound fertiliser at 750 kg·ha^−1^; (2) T123, CK23 + full straw return; (3) T223, CK23 + organic fertiliser (i.e., effective viable bacterial count ≥0.2 × 10^7^·g^−1^ and organic matter content ≥ 40%, Heilongjiang Wufu Min’an Agricultural Technology Co., Ltd., Qiqihar, China) at 450 kg·ha^−1^; (4) T323, compound fertiliser at 525 kg·ha^−1^ + full straw return; and (5) T423, compound fertiliser at 525 kg·ha^−1^ + organic fertiliser at 450 kg·ha^−1^. The organic fertiliser was applied at 450 kg·ha^−1^ and contained N ≥ 4% (i.e., approximately 18 kg N·ha^−1^), organic matter ≥ 40% and an effective viable bacterial count ≥0.2 × 10^7^·g^−1^; the C:N ratio of the organic material was approximately 10:1. These values were used as inputs to parameterise the organic matter decomposition and mineralisation module of WHCNS. Because this study focused on water and nitrogen cycling, model inputs and outputs were restricted to water and nitrogen balance terms, and other elements such as P and K were not considered. All fertilisers were applied once as basal fertiliser at sowing. The sowing date was 25 April, and the harvest date was 8 October.

### 4.3. Measurement Indicators and Methods

Soil moisture content and nitrate–nitrogen content were measured using the aluminium box method and a flow analyser (SEAL Analytical GmbH, Norderstedt, Germany), respectively, with three replicates per treatment. Measurements were conducted at the critical growth stages (i.e., seedling, jointing, silking, filling and maturity) over the 0–100 cm soil profile at 20 cm intervals. Plant dry matter mass was determined by first drying samples at 105 °C for 30 min for inactivation and then at 80 °C to constant weight. At maturity, yield was measured using three 1 m^2^ sample plots per treatment. Meteorological data were obtained from the Qiqihar Meteorological Station.

Water use efficiency (WUE) and System-Level Nitrogen Conversion Efficiency (SLNCE) were calculated using Equations (1) and (2), respectively, as follows:

WUE (kg·m^−3^) = Crop yield/Evapotranspiration (1)

SLNCE (kg·kg^−1^) = Crop yield/(Crop nitrogen uptake + Nitrogen leaching + Ammonia volatilisation + Denitrification) (2)

### 4.4. Overview and Validation of the WHCNS Model

The WHCNS model integrates modules from RZWQM, HYDRUS-1D and DAISY. It comprises seven submodules: meteorology, coupled soil water–heat–nitrogen transport, crop growth, field management, organic matter decomposition, root water–nitrogen uptake and inorganic nitrogen transformation. Together, these submodules quantitatively simulate water–carbon–nitrogen processes and their interactions within the soil–crop system [7,27]. The underlying principles and equations are described in detail by Liang et al. [7].

The WHCNS model was calibrated using the 2022 dataset and independently validated using the 2023 dataset. The parameters presented in Table 1 and Table 2 are the final calibrated values and were subsequently applied to the 2023 validation treatments. Simulation accuracy was evaluated using the normalised root mean square error (nRMSE), consistency index (*d*) and Nash–Sutcliffe efficiency coefficient (NSE) [28]. During calibration, the nRMSE, *d* and NSE values were 5.77–10.26%, 0.30–0.98 and 0.16–0.94 for soil water content; 11.52–20.32%, 0.78–0.98 and 0.41–0.92 for nitrate–nitrogen content (i.e., 0–60 cm); and 13.63–20.69%, 0.98–0.99 and 0.92–0.95 for aboveground dry matter mass, respectively. During validation, the corresponding nRMSE, *d* and NSE values were 6.01–17.14%, 0.24–0.96 and −3.02–0.83 for soil water content; 4.70–28.86%, 0.72–0.98 and −1.62–0.96 for nitrate–nitrogen content (i.e., 0–60 cm); and 7.08–11.22%, 0.97–0.99 and 0.94–0.98 for aboveground dry matter, respectively. Except for the marked deviation in soil water content for CK23 (NSE = −0.71) caused by extreme precipitation during the late growth stage, the simulated trends for all variables were highly consistent with the measured trends. These results indicate that the WHCNS model is applicable to maize fields in the semi-arid Chernozem region of Qiqihar [14] and can therefore be used for subsequent scenario simulations.

**Table 1:** Soil hydraulic parameters after calibration in the study area in 2023.

Soil Layer (cm)	Sand (%)	Silt (%)	Clay (%)	Unit Weight (g·cm^−3^)	Saturated Hydraulic Conductivity (cm·d^−1^)	Saturated Moisture Content (cm^3^·cm^−3^)	Residual Water Content (cm^3^·cm^−3^)	Soil Clay Content (cm^3^·cm^−3^)
0–20	17.8	29.7	52.5	1.43	10.18	0.469	0.097	0.523
20–40	19.6	29.1	51.3	1.56	4.92	0.420	0.091	0.516
40–60	21.3	29.1	49.6	1.68	3.060	0.398	0.085	0.495
60–80	22.7	29	48.3	1.74	2.230	0.370	0.081	0.484
80–100	23.5	28.4	48.1	1.79	1.68	0.353	0.078	0.480

**Table 2:** Calibration results for spring maize crop parameters for the T123 and T323 treatments.

Crop	Treatment	CC Initial	CC Middle	CC End	TEA Temperature (°C)	SLA (m^2^·kg^−1^) Maximum	SLA (m^2^·kg^−1^) Minimum	CO_2_ AR (kg·ha^−1^·h^−1^) Maximum	CO_2_ AR (kg·ha^−1^·h^−1^) Minimum	Maximum RL (cm)
Spring maize	T123	0.65	0.8	1.45	1700	25	15	63	0.52	120
Spring maize	T323	0.65	0.8	1.45	1700	27	14	59	0.39	120

Note: CC, Crop Coefficient; TEA, Total Effective Accumulated; SLA, Specific Leaf Area; AR, Assimilation Rate; RL, Root Length. These are the final calibrated parameters obtained from the 2022 dataset and subsequently used for the 2023 validation treatments (T123 and T323).

### 4.5. Evaluation of Model Simulation Performance

WHCNS model performance was evaluated using three metrics: (1) nRMSE, (2) *d* and (3) NSE. The nRMSE represents relative model deviation, with values closer to 0 indicating closer agreement between simulated and measured values. Accordingly, nRMSE values of <15%, 15–30% and >30% indicate excellent, good and poor model performance, respectively. The consistency index *d* evaluates predictive agreement and ranges from 0 to 1, with values closer to 1 indicating better agreement. NSE ranges from −∞ to 1, and values closer to 1 indicate better simulation performance. Based on the previously reported analysis [28], the proposed minimum thresholds for evaluating soil indicators under straw return conditions in the experimental area were *d* ≥ 0.75 and NSE ≥ 0, whereas the corresponding thresholds for crop growth were *d* ≥ 0.60 and NSE ≥ −1.0. The corresponding equations (Equations (3)–(6)) are as follows:

RMSE:

(3) $$\mathrm{RMSE}=\sqrt{\sum_{i=1}^{n}\frac{{\left(P_{i}\;-\;O_{i}\right)}^{2}}{n}}$$

*n*RMSE:

*n*RMSE = RMSE/*O* × 100% (4)

NSE:

(5) $$\mathrm{NSE}=1-\frac{\sum_{i=1}^{n}{\left(P_{I}\;-\;O_{I}\right)}^{2}}{\sum_{i=1}^{n}{\left(P_{I}\;-\;P_{\mathrm{avg}}\right)}^{2}}$$

*d*:

(6) $$d=1-\frac{\sum_{i=1}^{n}\frac{{\left(P_{i}\;-\;O_{i}\right)}^{2}}{n}}{\;{\sum_{i=1}^{n}\left(\left|P_{i}\;-\;O\right|\;+\;\left|O_{i}\;-\;O\right|\right)}^{2}}$$

where *P_i_* is the simulated value, *O_i_* is the measured value, *O* is the mean measured value, *n* is the total number of measured values and *P_avg_* is the mean simulated value.

### 4.6. Data Analysis

The WHCNS model was run independently for each of the three field replicates per treatment using the corresponding measured soil properties, initial conditions, crop parameters and field management inputs. Outputs from these three independent model runs (*n* = 3 per treatment) were treated as replicates for statistical analysis.

Normality and homogeneity of variances of the measured data (soil water content, nitrate–nitrogen content, aboveground dry matter and yield) were tested using the Shapiro–Wilk test and Levene’s test, respectively, prior to one-way ANOVA. Means of measured data were compared using Tukey’s HSD test at *p* < 0.05. Model-simulated outputs (Table 3, Table 4, Table 5 and Table 6) are reported as the mean ± standard deviation of three independent model runs per treatment (*n* = 3) and are presented as descriptive statistics only; no significance testing was applied to simulated values. Statistical analyses were performed using IBM SPSS Statistics 21.0 (version 21.0; IBM Corp., Armonk, NY, USA); figures were prepared using Origin 2018 (version 2018; OriginLab Corp., Northampton, MA, USA) and WPS Office (version 2010; Kingsoft Office, Beijing, China).

**Table 3:** WHCNS-simulated water balance components (mm) under different fertilisation treatments in 2023.

Crop	Treatment	Year	Rainfall (mm)	Irrigation (mm)	Evapotranspiration (mm)	Drainage Below 1 m (mm)	Water Balance Residual (mm)
Spring maize	CK23	2023	489.5	90	513.9	0	65.6
Spring maize	T123	2023	489.5	90	451.3	0	128.2
Spring maize	T223	2023	489.5	90	454.8	0	124.7
Spring maize	T323	2023	489.5	90	459.8	0	119.7
Spring maize	T423	2023	489.5	90	460.8	0	118.7

Note: Water Balance Residual = (Input − Output) = (Rainfall + Irrigation – Drainage − Evapotranspiration).

**Table 4:** WHCNS-simulated precipitation, evapotranspiration, crop yield and water use efficiency under different fertilisation treatments in 2023.

Crop	Treatment	Year	Precipitation (mm)	ET (mm)	Crop Yield (kg·ha^−1^)	WUE (kg m^−3^)
SM	CK23	2023.4.25–2023.10.8	579.5	513.9 ± 5.0	9556.1 ± 94.3	1.86 ± 0.02
SM	T123	2023.4.25–2023.10.8	579.5	451.3 ± 4.9	9412.5 ± 97.8	2.09 ± 0.02
SM	T223	2023.4.25–2023.10.8	579.5	454.8 ± 4.8	9745.9 ± 78.6	2.14 ± 0.03
SM	T323	2023.4.25–2023.10.8	579.5	459.8 ± 4.6	7854.0 ± 94.2	1.71 ± 0.01
SM	T423	2023.4.25–2023.10.8	579.5	460.8 ± 4.2	9041.4 ± 89.7	1.96 ± 0.02

Note: SM, Spring maize; ET, Evapotranspiration. WUE (kg·m^−3^) = Crop Yield (kg·ha^−1^)/[ET (mm) × 10]. Values are means ± standard deviations of three independent model runs per treatment (*n* = 3). Simulated outputs are presented as descriptive statistics; no significance test was applied.

**Table 5:** WHCNS-simulated ammonia volatilisation, crop uptake, leaching loss, denitrification and nitrogen use efficiency under different fertilisation treatments in 2023.

Crop	Treatment	Year	Ammonia Volatilisation (kg N ha^−1^)	Crop Uptake (kg N ha^−1^)	Leaching Loss (kg N ha^−1^)	Denitrification (kg N ha^−1^)	SLNCE (kg·kg^−1^)
SM	CK23	2023.4.25–2023.10.8	43.0 ± 0.4	221.2 ± 2.3	0	16.6 ± 0.2	34.0 ± 0.3
SM	T123	2023.4.25–2023.10.8	68.8 ± 0.7	260.5 ± 2.5	0	41.1 ± 0.4	30.8 ± 0.3
SM	T223	2023.4.25–2023.10.8	47.7 ± 0.5	219.7 ± 2.2	0	15.3 ± 0.2	34.5 ± 0.3
SM	T323	2023.4.25–2023.10.8	75.4 ± 0.8	173.9 ± 1.7	0	33.6 ± 0.3	27.8 ± 0.2
SM	T423	2023.4.25–2023.10.8	35.3 ± 0.4	201.8 ± 2.1	0	14.8 ± 0.1	35.9 ± 0.3

Note: SM, Spring maize.Values are means ± standard deviations of three independent model runs per treatment (*n* = 3). Simulated outputs are presented as descriptive statistics; no significance tests were applied.

**Table 6:** WHCNS-simulated ammonia volatilisation, crop uptake, leaching loss, denitrification and nitrogen use efficiency for the T323 treatment.

Treatment	Year	Precipitation (mm)	Leaching Loss (kg N ha^–1^)	Denitrification (kg N ha^–1^)	Ammonia Volatilisation (kg N ha^–1^)	Crop Uptake (kg N ha^–1^)	SLNCE
T323	2001	206.7	0	0.2 ± 0.1	34.4 ± 0.3	72.7 ± 0.7	19.5 ± 0.2
T323	2004	249.2	0	0.4 ± 0.1	35.2 ± 0.4	81.2 ± 0.8	20.2 ± 0.2
T323	2007	312.9	0	1.7 ± 0.1	35.5 ± 0.4	98.1 ± 1.0	18.6 ± 0.1
T323	2014	374.7	0	4.8 ± 0.1	36.1 ± 0.4	141 ± 1.5	26.1 ± 0.3
T323	2010	420.1	0	7.4 ± 0.1	36.3 ± 0.4	144.6 ± 1.4	33.5 ± 0.3
T323	2009	451.2	0	11.3 ± 0.1	36.5 ± 0.4	146.4 ± 1.5	33.5 ± 0.3
T323	2023	579.5	0	33.6 ± 0.3	75.4 ± 0.8	173.9 ± 1.7	27.8 ± 0.2

Note: Values are means ± standard deviations of three independent model runs per treatment (*n* = 3). Simulated outputs are presented as descriptive statistics; no significance test was applied.

## 5. Conclusions

Straw mulching and incorporation reduced evapotranspiration and increased WUE, supporting Hypothesis 2. Straw mulching alone produced the greatest reduction in evapotranspiration, whereas reduced chemical fertiliser combined with organic fertiliser maintained high yield and WUE. Reduced chemical fertiliser combined with organic fertiliser also increased system-level nitrogen conversion efficiency by improving N supply–demand synchrony, supporting Hypothesis 1.

Nitrate leaching below 1 m was negligible, whereas gaseous N loss dominated environmental N output. Gaseous N loss increased with precipitation and was more sensitive to the seasonal distribution of precipitation than to total precipitation.

The synergistic optimisation of WUE and system-level nitrogen conversion efficiency exhibited a threshold effect. Future dynamic water and nitrogen management strategies should therefore integrate multi-objective functions with climate year-type identification to address uncertainty in agricultural production under climate change.

Overall, both hypotheses were supported. Organic substitution improved N supply–demand synchrony and nitrogen conversion efficiency, whereas straw return reduced evapotranspiration and increased WUE; however, straw alone without supplemental N could not sustain yield or N efficiency. Three limitations should be recognised. First, WHCNS primarily simulates matrix flow and does not represent preferential flow through shrinkage cracks in clayey Chernozem soils. Second, the simulations included only water and nitrogen balance terms, whereas P and K were not considered. Third, the economic feasibility of large-scale organic fertiliser adoption was not quantified. Future work should therefore combine multi-objective optimisation, real-time soil moisture and nitrogen monitoring and comprehensive economic assessment across different precipitation-year types.

## Data Availability

Data are contained within the article.
